# Spontaneous Regression of Soft Tissue Sarcoma Following Biopsy: A Case Report and Systematic Review of the Literature

**DOI:** 10.7759/cureus.107111

**Published:** 2026-04-15

**Authors:** Megan C Gannon, Rachel M Gabor, Anushka Gupta, Cheshta Gupta, Ruhi M Shah, Rohit Sharma

**Affiliations:** 1 Department of Surgery, Marshfield Clinic Health System, Marshfield, USA; 2 Center for Clinical Epidemiology and Population Health, Marshfield Clinic Research Institute, Marshfield Clinic Health System, Marshfield, USA; 3 Department of Physiology and Medical Cell Biology, Western University, London, CAN

**Keywords:** low-grade myxofibrosarcoma, soft-tissue sarcoma, spontaneous tumor regression, systematic review and meta analysis, upper extremity sarcoma

## Abstract

Spontaneous regression (SR) of malignancy is a rare phenomenon offering unique insights into tumor-host immunology. In sarcomas, the incidence, triggers, and clinical implications of SR are poorly characterized. We aim to synthesize the published literature on SR in sarcoma and report a novel case from our institution where diagnostic biopsy triggered a complete pathological response (pCR).

We retrospectively reviewed one institutional case: a 59-year-old female with myxofibrosarcoma (MFS) who demonstrated significant regression following core needle biopsy. We subsequently conducted a descriptive systematic review in accordance with Preferred Reporting Items for Systematic Reviews and Meta-Analyses (PRISMA) guidelines, rather than a formal meta-analysis. PubMed, Embase, and Scopus were searched from inception to July 2025 for reports of SR in pathologically confirmed sarcomas without prior cytotoxic therapy.

In our institutional experience, the patient (59-year-old female, MFS) achieved complete clinical resolution within weeks of biopsy. Definitive wide excision revealed no viable tumor cells, confirming pCR. The systematic review identified 32 published studies describing 32 unique cases of SR in sarcoma. Diagnostic biopsy was the leading identified trigger (25%). Biopsy-associated regressions occurred more rapidly (median 0.9 months) compared to infection-associated (median five months) or spontaneous cases. Despite clinical regression, 75% of patients in the biopsy-triggered group underwent definitive surgical resection.

SR in sarcoma is frequently preceded by an immune-stimulating event, most notably biopsy. While SR provides in vivo evidence of host anti-tumor immunity, the potential for incomplete or transient response underscores that SR should not preclude definitive oncologic management. Ultimately, SR can be conceptualized as a "neoadjuvant immune boost" rather than a definitive cure. Wide surgical resection based on pre-regression tumor fields remains the standard of care to eradicate microscopic disease and ensure oncologic safety.

## Introduction

Soft tissue and bone sarcomas constitute a heterogeneous group of rare mesenchymal malignancies, accounting for approximately 1% of all adult cancers [1]. Encompassing over 70 histological subtypes, these tumors exhibit diverse clinical behaviors. Myxofibrosarcoma (MFS) is one of the most common subtypes in older adults, characterized by infiltrative growth and a high propensity for local recurrence [2]. For localized disease, the standard of care remains wide surgical resection, often supplemented by radiation therapy to ensure local control [3].

Spontaneous regression (SR), defined by Everson and Cole as the partial or complete disappearance of a malignant tumor in the absence of specific oncologic therapy, is an exceptionally rare biological event, estimated to occur in one out of every 60,000 to 100,000 cancer cases [4]. While documented in immunogenic tumors like melanoma and renal cell carcinoma, reports of SR in sarcoma are sporadic and largely anecdotal. Understanding the mechanisms of SR is of critical clinical interest. In an era of emerging immunotherapies, cases of SR may represent "natural models" of effective host anti-tumor immunity. However, the phenomenon also presents a clinical dilemma: when a sarcoma shrinks unexpectedly, does this obviate the need for surgery, or is it a temporary lull in an aggressive disease?

This study aims to address this knowledge gap. We report a remarkable case of complete pathological response (pCR) in a high-grade sarcoma following diagnostic biopsy and present a comprehensive descriptive systematic review of the literature to characterize the triggers, pathological features, and optimal management of this rare phenomenon.

## Case presentation

Institutional case report: myxofibrosarcoma of the right forearm

We retrospectively reviewed the clinical course, imaging, and pathological records of a patient who was managed at the Marshfield Clinic Health System in Marshfield, Wisconsin, USA, with SR of sarcoma. Written informed consent was obtained from the patient for the publication of this case and accompanying images.

**Figure 1:**
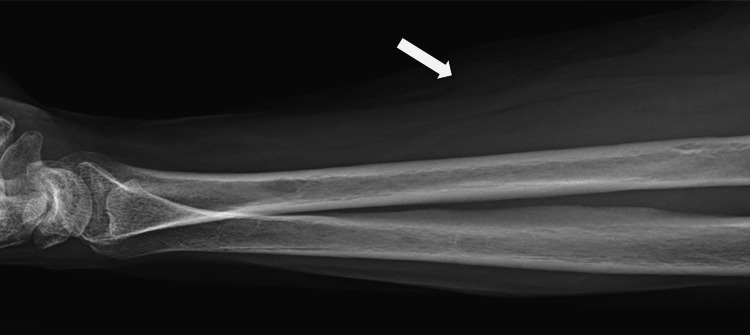
X-ray of the right forearm Soft tissue density along the mid-volar forearm (white arrow).

A 59-year-old female presented with a rapidly enlarging mass in the right upper extremity. Physical examination revealed a 2 x 2 cm palpable, firm, semi-mobile lesion on the volar aspect of the right forearm. Motor and sensory functions were entirely intact, with no overlying skin changes. Laboratory investigations were within normal limits (e.g., hemoglobin: 12.1 g/dL (reference: 12.0-15.5 g/dL), white blood cell count: 6.3 x 10^9/L (reference: 4.5-11.0 x 10^9/L)). The initial right forearm X-ray demonstrated a non-specific soft tissue density (Figure 1).

**Figure 2:**
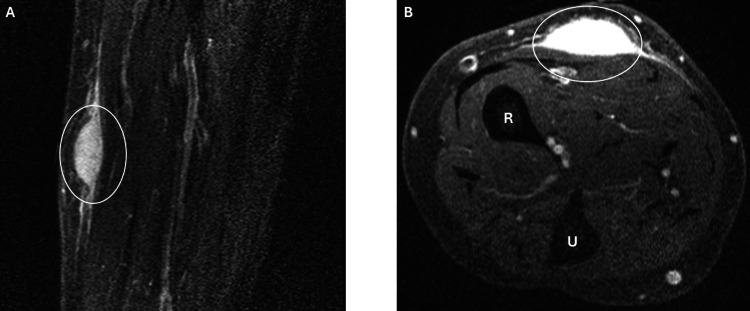
Magnetic resonance imaging (MRI) of the right radius/ulna A 1.9 cm diffuse enhancing ovoid soft tissue mass within the volar radial mid right forearm subcutaneous tissues, concerning for peripheral nerve sheath tumor of the peripheral nerve branch, sarcoma, or malignant fibrous histiocytoma. A) T1-weighted sagittal ovoid soft tissue mass (white circle); B) T1-weighted axial ovoid soft tissue mass (white circle), radius (R), and ulna (U).

Subsequent MRI demonstrated a 1.9 cm diffuse enhancing ovoid soft tissue mass consistent with sarcoma (Figure 2A, 2B).

**Figure 3:**
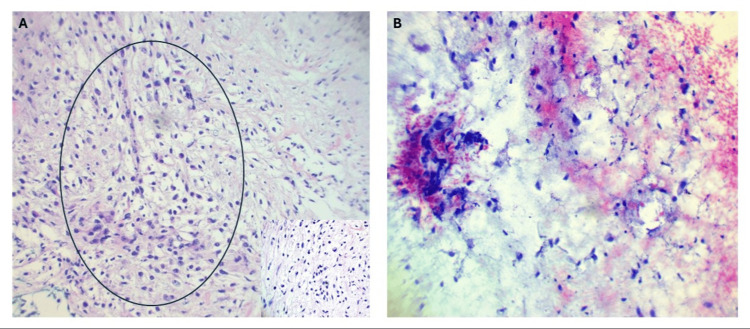
Core needle biopsy and fine needle aspirate of the right forearm myxofibrosarcoma A) Core needle biopsy: atypical spindle cells with nuclear hyperchromasia in a fibromyxoid background, with condensation of neoplastic cells around curvilinear capillaries (within the black circle). Higher magnification: atypical spindle cells with angulated hyperchromatic nuclei in a myxoid background. B) Fine needle aspirate: highlights the tumor’s myxoid background.

A core needle biopsy and fine needle aspirates were performed, and histopathology confirmed an intermediate-grade MFS (Figure 3A, 3B).

**Figure 4:**
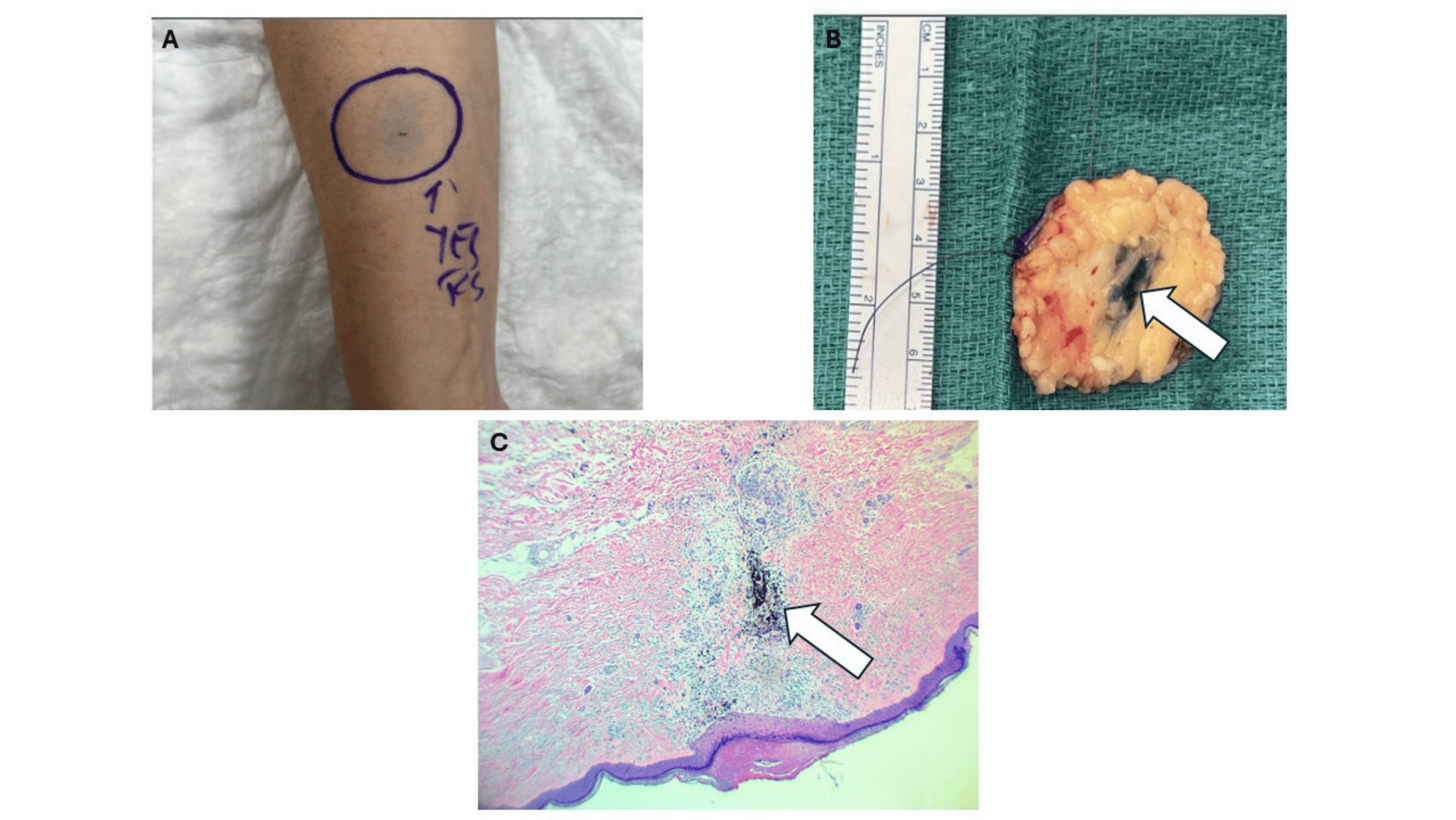
Myxofibrosarcoma excision and final pathology A) Pre-operative marking of right forearm sarcoma with visible ink tattoo; B) Intra-op right forearm sarcoma, excision: right forearm subcutaneous tissue with a healing biopsy scar containing tattoo ink (white arrow), no residual sarcoma identified; C) Final pathology: dermal cicatrix (consistent with previous needle biopsy site), harboring India ink tattoo (white arrow). These are original diagnostic and pathological images from the patient described in this report, and written informed consent was obtained for publication.

Following the biopsy, the patient noted a rapid decrease in the size of the right forearm mass. Within two weeks, the lesion was no longer palpable, demonstrating a complete clinical response. Despite the clinical resolution, the decision was made to proceed with oncologic resection to ensure disease control. The patient underwent a wide local excision of the tumor bed. Detailed pathological examination of the resected specimen revealed extensive fibrosis and inflammatory changes but no viable tumor cells, consistent with a pCR (Figure 4A-4C). The patient remains disease-free at the one-year follow-up.

## Discussion

Systematic review methodology

A systematic search of PubMed, Embase, and Scopus databases was performed from inception to July 2025 in accordance with Preferred Reporting Items for Systematic Reviews and Meta-Analyses (PRISMA) guidelines. The search strategy utilized Boolean operators combining sarcoma-specific terms (e.g., "Sarcoma," "Myxofibrosarcoma," "Angiosarcoma," "Osteosarcoma") with regression terms (e.g., "Spontaneous Regression," "Spontaneous Remission," "Spontaneous Resolution").

Studies were included if they reported: pathologically confirmed sarcoma; objective tumor regression documented by imaging or clinical exam; and absence of prior cytotoxic chemotherapy, targeted molecular therapy, or definitive radiation. Cases of "sarcoma-like" reactive processes (e.g., nodular fasciitis) were excluded. Data regarding patient demographics, histology, potential triggers (e.g., biopsy, infection), time to regression, management (observation vs. surgery), and final outcomes were extracted.

The systematic review analysis was strictly descriptive, utilizing non-parametric tests (Mann-Whitney U, Kruskal-Wallis, Fisher's exact test) to compare median kinetic values across trigger groups where appropriate. All analysis was completed in R version 4.4.1 (R Core Team, Vienna, Austria, 2025). 

Systematic review findings

**Figure 5:**
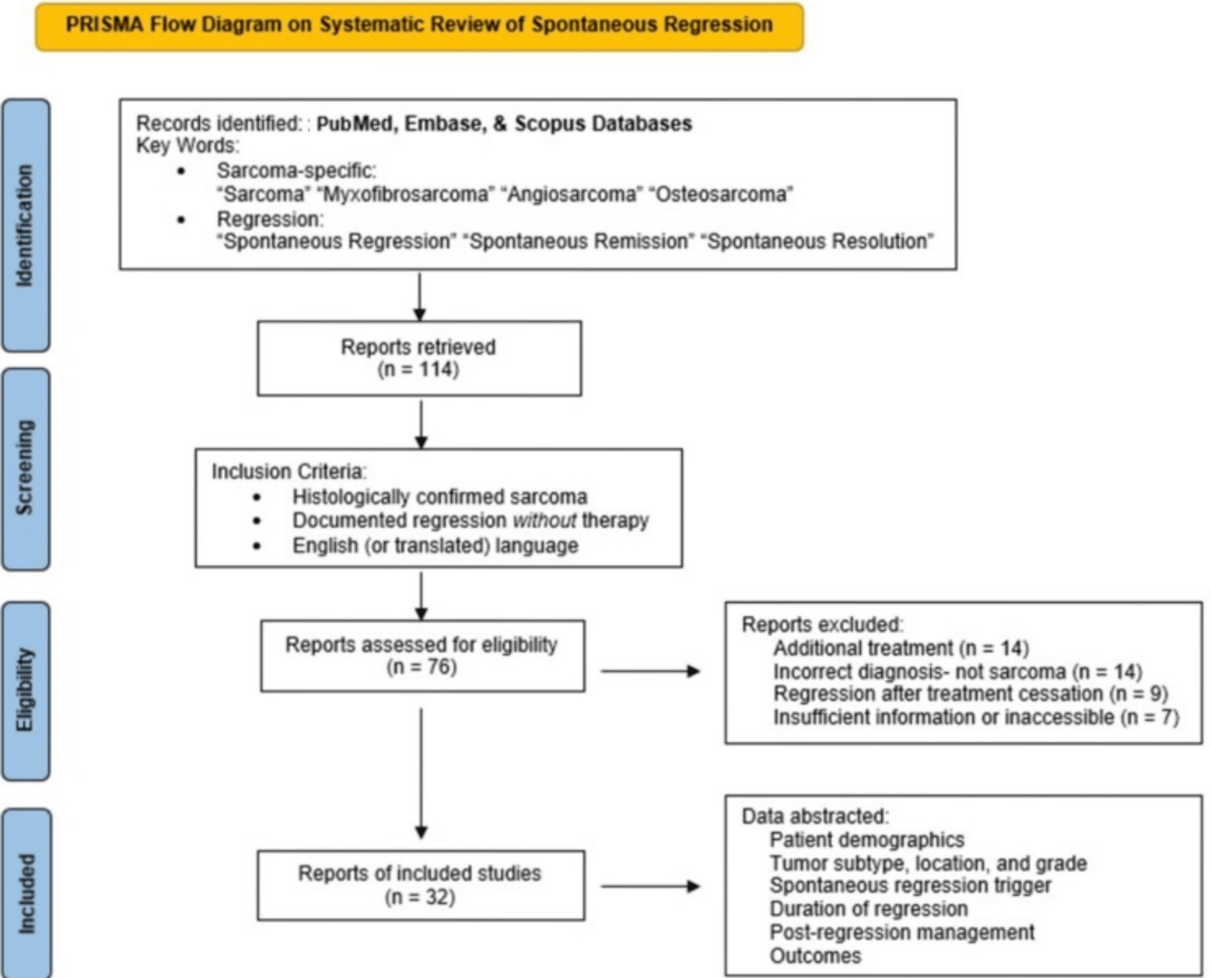
PRISMA flow diagram of the systematic review of spontaneous regression PRISMA: Preferred Reporting Items for Systematic Reviews and Meta-Analyses

The systematic literature search yielded 114 potential articles. After screening for inclusion criteria, 32 studies describing 32 unique cases of SR in sarcoma were included for analysis (Figure 5) [2-33].

Demographics and Tumor Characteristics

The median age of patients in the literature was 50 years (range 0-85). There was a female predominance, with 59.4% (19/32 reporting) of patients being female. The cohort was histologically diverse, with reported subtypes including angiosarcoma, infantile fibrosarcoma, osteosarcoma, and MFS.

Triggers of Regression

A potential inciting event preceding regression was identified in the majority of cases. Diagnostic biopsy/trauma was the leading identified trigger, noted in 25% (8/32) of cases. Systemic infections (e.g., pneumonia, sepsis) were identified in approximately 9% (3/32) of cases. In the remaining cases, no specific trigger was identified, or rare triggers (e.g., cessation of immunosuppression) were noted.

Kinetics of Regression

The systematic review revealed distinct differences in the time course of regression based on the trigger. Regression in the biopsy-triggered group occurred rapidly, with a median time from trigger to regression of 0.9 months (range 0.3-5 months). In contrast, regression associated with infection took a median of five months, and regression associated with spontaneous/other causes took a median of three months (p=0.140).

Management and Outcomes

In the biopsy-triggered group, 50% (4/8) of patients achieved complete regression, while 25% achieved near-complete and 25% partial regression. Despite observing significant tumor shrinkage, definitive surgical resection was performed in 75% (6/8) of the biopsy-triggered cases. Pathologically, in the biopsy group where surgery was performed, 50% of cases showed no residual tumor (pCR), while 37.5% showed persistent microscopic disease, highlighting the risk of observation alone.

The "Biopsy Effect" and Immune Activation

This study highlights the rare but distinct phenomenon of biopsy-induced SR in soft tissue sarcoma. Our institutional case, an intermediate-grade MFS, followed a striking clinical course: rapid, complete regression initiated immediately after core needle biopsy.

Our findings align with the systematic review data, which identified biopsy as the leading trigger for SR (25%). We hypothesize that the mechanical disruption of the tumor architecture during biopsy releases tumor-associated antigens (TAAs) into the circulation while simultaneously creating a local inflammatory wound-healing environment [5]. This combination may break local immune tolerance, recruiting antigen-presenting cells and priming cytotoxic T-cells to mount a targeted response [6]. The pathological findings in our case, fibrosis and inflammation replacing the tumor, mirror the findings in literature cases like those by Mizuno et al., providing histological evidence for an immune-mediated mechanism [7].

Time Course and Kinetics

Our review suggests that biopsy-induced regression occurs rapidly (median 0.9 months) compared to infection-associated regression (median five months). While this difference did not reach statistical significance (p=0.140), likely due to the small sample size inherent to this rare phenomenon, the rapid kinetics support an acute inflammatory/immune mechanism rather than a slow hormonal or metabolic adjustment. This finding is clinically relevant; if regression is going to occur post-biopsy, it will likely present before the standard window for definitive surgery (typically four to six weeks).

Clinical Implications: The Danger of "Watch and Wait"

The observation of a regressing sarcoma presents a clinical trap. It may tempt the clinician to cancel surgery. However, our systematic review reveals that while some regressions are complete, many are partial or transient. Furthermore, in the biopsy group, nearly 40% of resected specimens still contained residual tumor cells despite clinical regression.

Crucially, in our institutional case, definitive surgery was performed despite clinical resolution. This provided the necessary pathological confirmation of cure. Had this patient been observed, the status of the tumor bed would have remained unknown. Therefore, we advocate that SR should be viewed as a "neoadjuvant immune boost." The standard of care-wide surgical resection based on pre-regression tumor boundaries must be maintained to ensure the eradication of microscopic disease.

Limitations

This study has several limitations. Most notably, there is an inherent publication bias toward successful regression cases, as instances of non-regression following biopsy are not typically published as case reports. Additionally, the heterogeneity of over 70 distinct sarcoma subtypes limits the ability to generalize immunological mechanisms across all histologies, as various subtypes may respond differently to immune triggers and mechanical perturbations.

## Conclusions

SR in sarcoma is a rare phenomenon frequently catalyzed by an immune-stimulating event such as a diagnostic biopsy. We report a case of complete pathological regression following biopsy, adding to a growing body of evidence that mechanical perturbation can trigger effective anti-tumor immunity. However, given the aggressive nature of sarcomas and the potential for residual microscopic disease, the observation of SR should not deter clinicians from pursuing definitive surgical resection to ensure oncologic safety.
